# Small Molecule Analysis of Extracellular Vesicles Produced by *Cryptococcus gattii*: Identification of a Tripeptide Controlling Cryptococcal Infection in an Invertebrate Host Model

**DOI:** 10.3389/fimmu.2021.654574

**Published:** 2021-03-16

**Authors:** Flavia C. G. Reis, Jonas H. Costa, Leandro Honorato, Leonardo Nimrichter, Taícia P. Fill, Marcio L. Rodrigues

**Affiliations:** ^1^Instituto Carlos Chagas, Fundação Oswaldo Cruz (Fiocruz), Curitiba, Brazil; ^2^Centro de Desenvolvimento Tecnológico em Saúde (CDTS), Fiocruz, Rio de Janeiro, Brazil; ^3^Institute of Chemistry, University of Campinas, Campinas, Brazil; ^4^Instituto de Microbiologia Paulo de Góes (IMPG), Universidade Federal do Rio de Janeiro, Rio de Janeiro, Brazil

**Keywords:** *Cryptococcus gatti*, extracellular vesicles, small molecules, mass spectrometry, *Galleria mellonella*

## Abstract

The small molecule (molecular mass <900 Daltons) composition of extracellular vesicles (EVs) produced by the pathogenic fungus *Cryptococcus gattii* is unknown, which limits the understanding of the functions of cryptococcal EVs. In this study, we analyzed the composition of small molecules in samples obtained from solid cultures of *C. gattii* by a combination of chromatographic and spectrometric approaches, and untargeted metabolomics. This analysis revealed previously unknown components of EVs, including small peptides with known biological functions in other models. The peptides found in *C. gattii* EVs had their chemical structure validated by chemical approaches and comparison with authentic standards, and their functions tested in a *Galleria mellonella* model of cryptococcal infection. One of the vesicular peptides (isoleucine-proline-isoleucine, Ile-Pro-Ile) improved the survival of *G. mellonella* lethally infected with *C. gattii* or *C. neoformans*. These results indicate that small molecules exported in EVs are biologically active in *Cryptococcus*. Our study is the first to characterize a fungal EV molecule inducing protection, pointing to an immunological potential of extracellular peptides produced by *C. gattii*.

## Introduction

*Cryptococcus gattii* is a fungal pathogen that causes disease in immunocompetent individuals. This fungus was responsible for outbreaks in the Pacific Northwest and in the Vancouver Island (1). *C. gattii* virulent strains, which are endemic in Brazil (2), likely emerged from South America (3). *C. gattii* can cause severe lung disease and death without dissemination. In contrast, its sibling species *C. neoformans* disseminates readily to the central nervous system (CNS) and causes death from meningoencephalitis (1). *C. gattii* and *C. neformans* share major virulence determinants, including the ability to produce extracellular vesicles (EVs) (4–6). EVs are membranous structures produced by prokaryotes and eukaryotes, including 14 fungal genera (7). In fungi, they were first characterized in culture fluids of *C. neoformans* (6). A decade later, *C. gattii* was also demonstrated to produce EVs in liquid matrices (4).

The perception that EVs are essential players in both physiology and pathogenesis of fungi is now consolidated. Much of the knowledge on the functions of fungal EVs has derived from studies of their composition. During the last decade, proteins, lipids, glycans, and nucleic acids were characterized as components of fungal EVs (8, 9). Molecules of low molecular mass, however, have been overlooked. Two recent studies have characterized the small molecule composition of *Histoplasma capsulatum* (10) and *Penicillium digitatum* EVs (11), but the low molecular mass components of other fungal EVs are unknown. Considering the molecular diversity found in both *H. capsulatum* and *P. digitatum*, it is plausible to predict that many still unknown functions of EV components of low molecular mass remain to be characterized. In fact, the metabolome analysis of *P. digitatum* EVs revealed the presence of phytopathogenic molecules that inhibited the germination of the plant host's seeds (11).

We have recently described a protocol for the isolation of cryptococcal EVs through which the vesicles were obtained from solid fungal cultures (5). Although the general properties of fungal EVs obtained from solid cultures resembled those described for vesicles obtained from liquid media, a recent analysis of the protein composition of cryptococcal EVs obtained from solid medium revealed important differences in comparison to those obtained in early studies using liquid cultures (12, 13). This observation and the fact that culture conditions impact the composition of small molecules in *H. capsulatum* EVs (10) reinforce the importance of the compositional characterization of vesicles obtained from solid medium.

In this manuscript, we characterized the low mass components of EVs produced by *C. gattii*. The synthesis of some of the small molecules detected in the EVs revealed a vesicular peptide that protected an invertebrate host against a lethal challenge with *C. gattii* in a dose-dependent fashion. These results indicate the existence of new venues of exploration of the functions of EVs in fungal pathogens, and suggest that small molecules of fungal EVs have immunological potential.

## Results

### Small Molecule Characterization of *C. gattii* EVs

**Figure 1:**
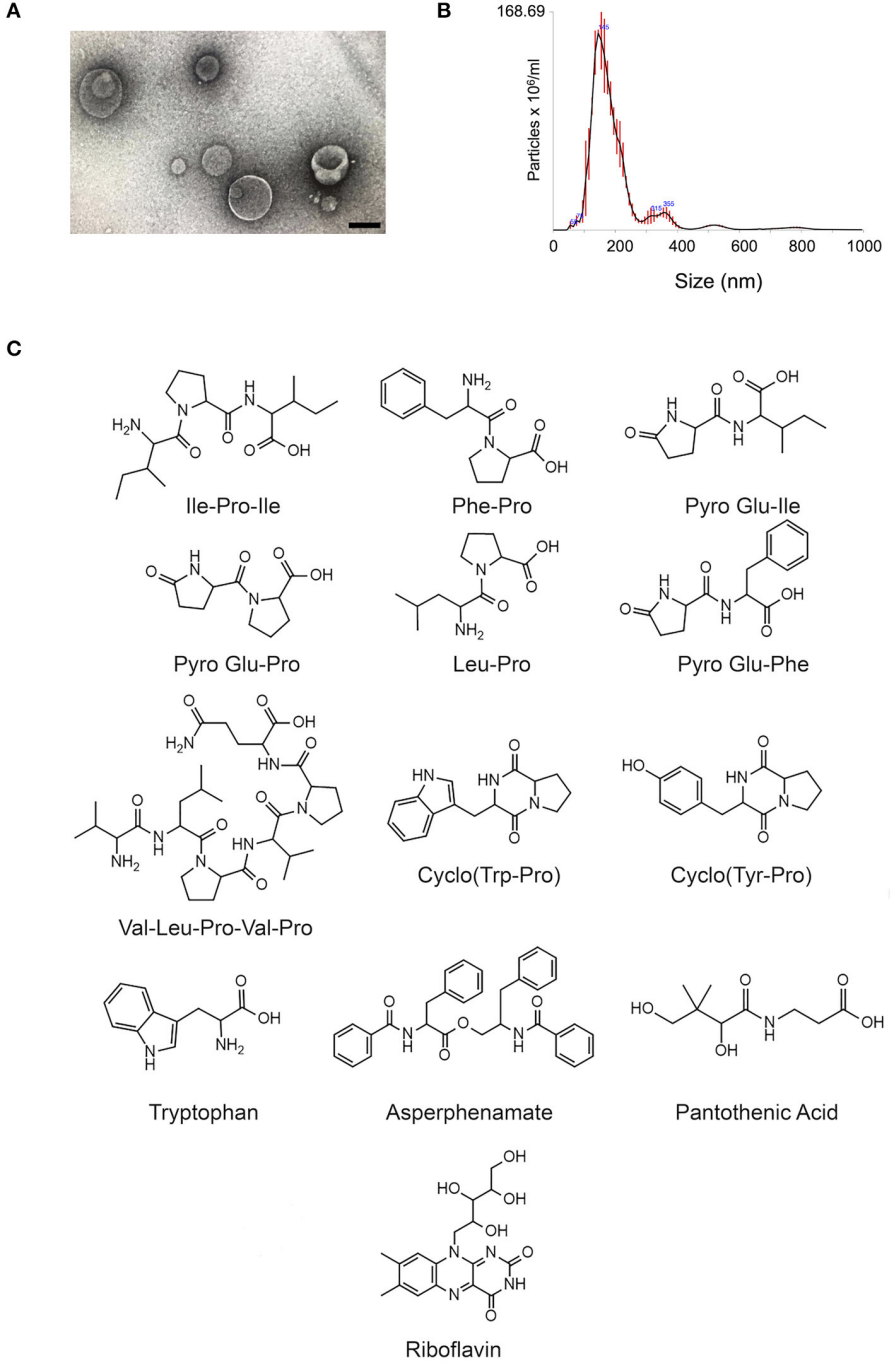
Small molecule characterization in EVs produced by *C. gattii*. **(A)**. Transmission electron microscopy analysis (negative staining) of a *C. gattii* EV sample submitted to chemical characterization by mass spectrometry. Scale bar, 100 nm. **(B)**. Nanoparticle tracking analysis of EVs obtained from *C. gattii* cultures, showing the typical distribution of EVs in the 50–250 nm range, and a minor population in the 300–400 nm range. EVs shown in **(A,B)** illustrate the characteristics found in three independent samples with similar results. **(C)**. Structures of the metabolites identified in *C. gatti* EVs through the GNPS MS/MS database. Amino acid codes represent isoleucine (Ile), proline (Pro), phenylalanine (Phe), glutamic acid (Glu), leucine (Leu), valine (Val), tryptophan (Trp), and tyrosine (Tyr).

*C. gatti* EV samples (Figures 1A,B) were prepared as independent triplicates. EV extracts were analyzed by ultra-high performance liquid chromatography-tandem mass spectrometry (UHPLC-MS/MS), and the data submitted to molecular networking analysis in the Global Natural Product Social Molecular Networking (GNPS) platform, an interactive online small molecule–focused tandem mass spectrometry data curation and analysis infrastructure (14). Molecular networking using high-resolution MS/MS spectra allows the organization of vesicular compounds in a visual representation (15, 16). In this analysis, each node is labeled by a precursor mass and represents a MS/MS spectrum of a compound, and compounds of the same molecular family are grouped together, connected by arrows, forming clusters of similarity (15–18). Since the molecules can be identified in a database through their fragmentation patterns and are represented in the molecular networking, the benefits of this approach include fast dereplication, identification of similar compounds, and effortless comparisons between different metabolic profiles or conditions (16, 17).

**Figure 2:**
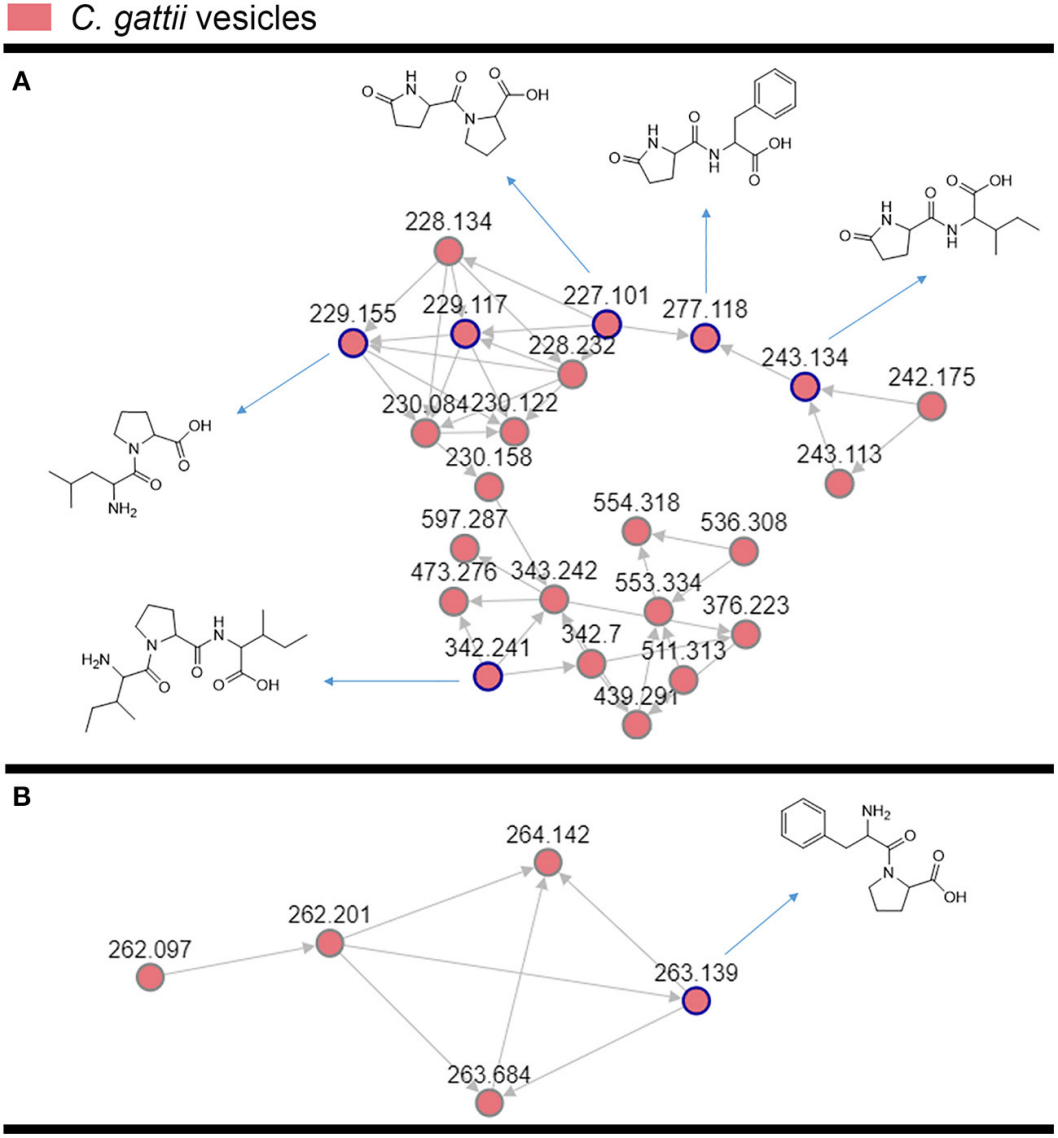
Molecular networking visualization of the peptides identified in the *C. gattii* EV cargo. Clusters **(A,B)** were obtained through molecular networking analysis of the components of *C. gattii* EVs, which were obtained after organic extraction of secondary metabolites. Each node is represented as a compound's MS/MS spectrum. Blue nodes are represented as spectra of identified constituents obtained by comparison with the GNPS platform database. The peptides identified in the GNPS platform had their identity further confirmed by comparison with synthetic standards. The gray nodes represent the spectra of their unknown analogs with similar fragmentation patterns.

The cluster-based molecular networking analysis revealed secondary metabolites present in the *C. gattii* EVs. The molecules detected in our analysis were classified as EV components if they were detected in the three replicates. Using this criterion, our small molecule analysis identified 13 genuine components of the *C. gattii* EV samples (Table 1). This analysis revealed previously unknown components of EVs, including peptides, amino-acids, vitamins, and a carboxylic ester. The metabolites were identified through hits in the GNPS database (Supplementary Figures 1–13) and corresponded to Ile-Pro-Ile (*m/z* 342.2384), Phe-Pro (*m/z* 263.1387), pyro Glu-Ile (*m/z* 243.1335), pyro Glu-Pro (*m/z* 227.1022), Leu-Pro (*m/z* 229.1544), pyro Glu-Phe (*m/z* 277.1180), Val-Leu-Pro-Val-Pro (*m/z* 652.4025), cyclo (Trp-Pro) (*m/z* 284.1393), cyclo (Tyr-Pro) (*m/z* 261.1234), tryptophan (*m/z* 205.0972), asperphenamate (*m/z* 507.2278), riboflavin (*m/z* 377.1456), and pantothenic acid (*m/z* 220.1181). The structures and MS data of the detected metabolites are shown in Figure 1C, Table 1, respectively. The cluster-based molecular networking analysis of the *C. gattii* EV components is detailed in Figure 2.

**Table 1:** MS data obtained for *Cryptococcus gattii* secondary metabolites detected on EVs.

**Compound**	**Ion formula**	**Calculated** ***m/z***	**Experimental** ***m/z***	**Error (ppm)**
Ile-Pro-Ile (Diprotin A)	C_17_H_32_N_3_O_4_	342.2392	342.2384	−1.1
Phe-Pro	C_14_H_19_N_2_O_3_	263.1395	263.1387	−1.3
Pyro-Glu-Ile	C_11_H_19_N_2_O_4_	243.1344	243.1335	−1.8
Pyro-Glu-Pro	C_10_H_15_N_2_O_4_	227.1031	227.1022	−1.7
Leu-Pro	C_11_H_21_N_2_O_3_	229.1552	229.1544	−1.4
Pyro-Glu-Phe	C_14_H_17_N_2_O_4_	277.1188	277.1180	−1.0
Val-Leu-Pro-Val-Pro	C_31_H_54_N_7_O_8_	652.4033	652.4025	−1.2
Cyclo(Trp-Pro)	C_16_H_18_N_3_O_2_	284.1399	284.1393	−2.1
Cyclo(Tyr-Pro)	C_14_H_17_N_2_O_3_	261.1239	261.1234	−1.9
Tryptophan	C_11_H_13_N_2_O_2_	205.0977	205.0972	−2.4
Asperphenamate	C_32_H_31_N_2_O_4_	507.2283	507.2278	−1.0
Riboflavin	C_17_H_21_N_4_O_6_	377.1461	377.1456	−1.3
Pantothenic acid	C_9_H_18_NO_5_	220.1184	220.1181	−1.3

**Figure 3:**
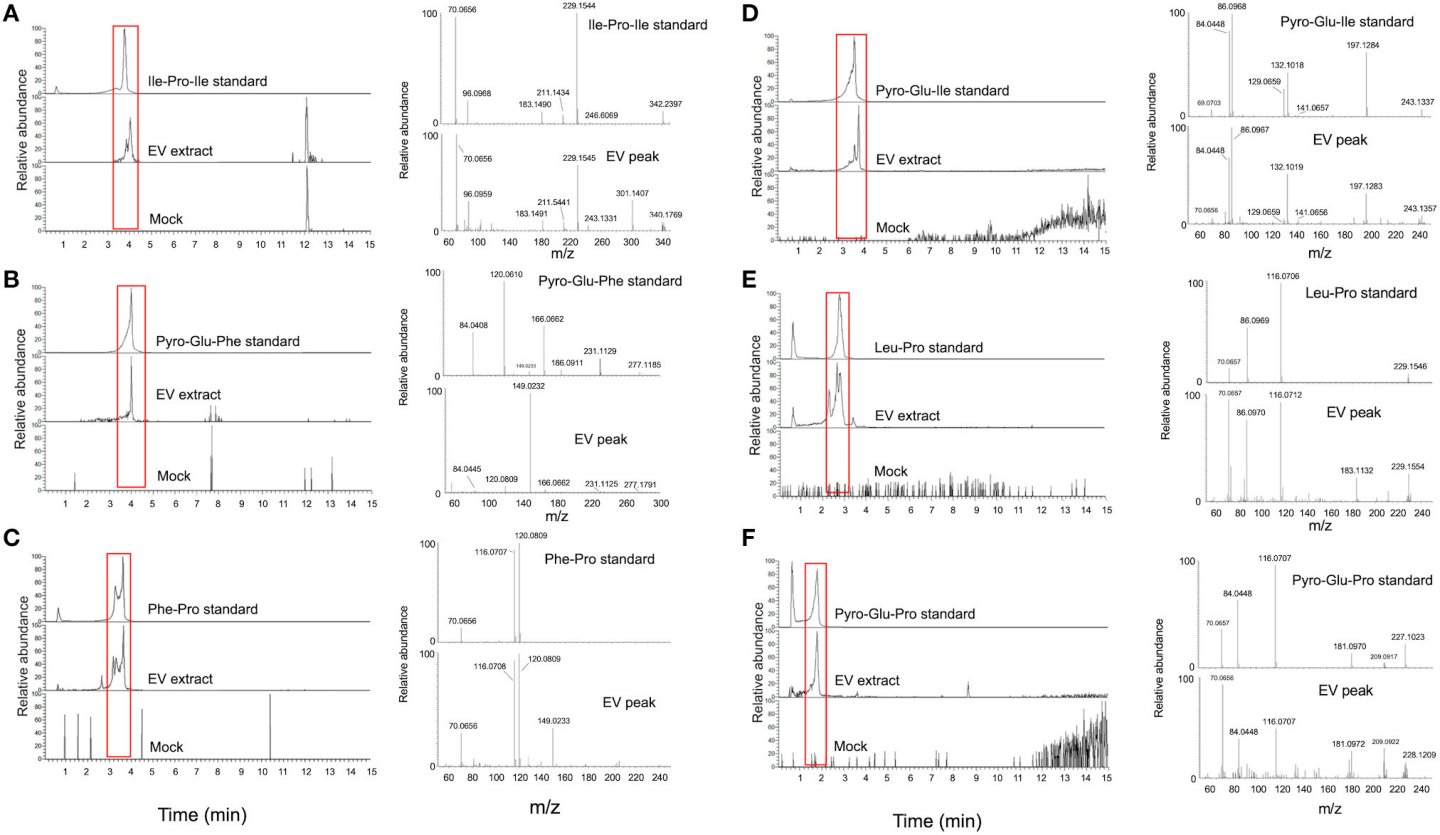
Structural analysis of EV peptides produced by *C. gattii*, including Ile-Pro-Ile **(A)**, pyro-Glu-Phe **(B)**, Phe-Pro **(C)**, pyro-Glu-Ile **(D)**, Leu-Pro **(E)**, and pyro-Glu-Pro **(F)**. For each peptide, the chromatographic separation of synthetic standards, EV extracts, and control (mock) samples is presented on the left side of each panel. The peaks with retention times similar to the corresponding standards (red boxed area) were selected for fragmentation by mass spectrometry (MS). The MS fragmentation profiles are shown on the right side of each panel. These analyses confirmed that the structural match between the EV components and the synthetic standards.

For validation of some key GNPS hits, we performed another round of spectrometric characterization of *C. gattii* small molecules including additional criteria as follows. We classified as authentic EV compounds those whose structure was observed in EV extracts, but not in mock samples (extracted from sterile culture medium). Finally, these compounds obligatorily had chromatographic and spectrometric properties similar to those of synthetic standards. Due to the easiness in chemical synthesis and lack of detailed information in the literature, the linear dipeptides Phe-Pro, pyro-Glu-Ile, pyro-Glu-Pro, Leu-Pro, and pyro-Glu-Phe, and the tripeptide Ile-Pro-Ile were selected for the validation assays. We then searched for their presence in EV and mock extracts. The six peptides selected for chemical synthesis were classified as authentic EV components according to these criteria (Table 2). Indeed, this analysis revealed similar fragmentation patterns and retention times for the vesicle peptides and the standard metabolites (Figure 3). The peptides exhibited typical fragments of protonated amino acids at *m/z* 70.06, 86.09, 116.07, and 120.08 (Supplementary Figure 14). In compounds containing proline, fragments at *m/z* 116.07 and 70.06 corresponded, respectively, to the loss of protonated proline and subsequent loss of H_2_O and CO. In peptides composed by isoleucine or leucine, fragments at *m/z* 132.02 and 86.09 corresponded, respectively, to protonated leucine/isoleucine and subsequent loss of H_2_O and CO. Finally, the loss of H_2_O and CO in protonated phenylalanine formed the major fragment ion at *m/z* 120.08 (19). Assuming that the vesicular components are synthesized within the cells and exported extracellularly, we also analyzed cellular and supernatant extracts. The six peptides listed in Table 2 were also found in these extracts (data not shown). These results were highly reproducible. Of note, the analysis of the presence of the peptides listed in Table 2 was performed independently by two laboratory members in 12 EV samples produced by three different strains of *C. gattii*. All six peptides were found in all assays (data not shown).

**Table 2:** Chromatographic identification of peptides in cryptococcal EVs^*^.

**Peptide**	**Control**	**Mock**	**EVs**	**Synthetic standards**
Ile-Pro-Ile	NF	NF	4.03	3.76
Phe-Pro	NF	NF	3.18 and 3.64	3.28 and 3.62
Pyro-Glu-Ile	NF	NF	3.55	3.53
Pyro-Glu-Pro	NF	NF	1.8	1.8
Leu-Pro	NF	NF	2.83	2.78
Pyro-Glu-Phe	NF	NF	4	4

*Sample (retention time, min)*.

* *Peptide identification was performed in blank samples (control) in addition to preparations obtained from sterile medium (mock) or fungal EVs. The results were compared to those obtained with synthetic peptides. NF, not found*.

### Biological Activity of EV Peptides of *C. gattii*

After characterization of Ile-Pro-Ile, Phe-Pro, pyro-Glu-Ile, pyro-Glu-Pro, Leu-Pro, and pyro-Glu-Phe as authentic EV components of *C. gattii*, we used their synthetic forms to analyze their possible biological activities. On the basis of the previously reported ability of fungal peptides to kill bacteria (20), we initially tested their antibacterial capacity against *Staphylococcus aureus* and *Pseudomonas aeruginosa*. None of the peptides had any effect on microbial growth (data not shown). Since cryptococcal EVs regulate intercellular communication (4), we also speculated that the peptides could mediate quorum sensing, Titan cell formation, or capsule growth. Once again, none of the peptides had any apparent effects on these processes in *C. gattii* (data not shown).

**Figure 4:**
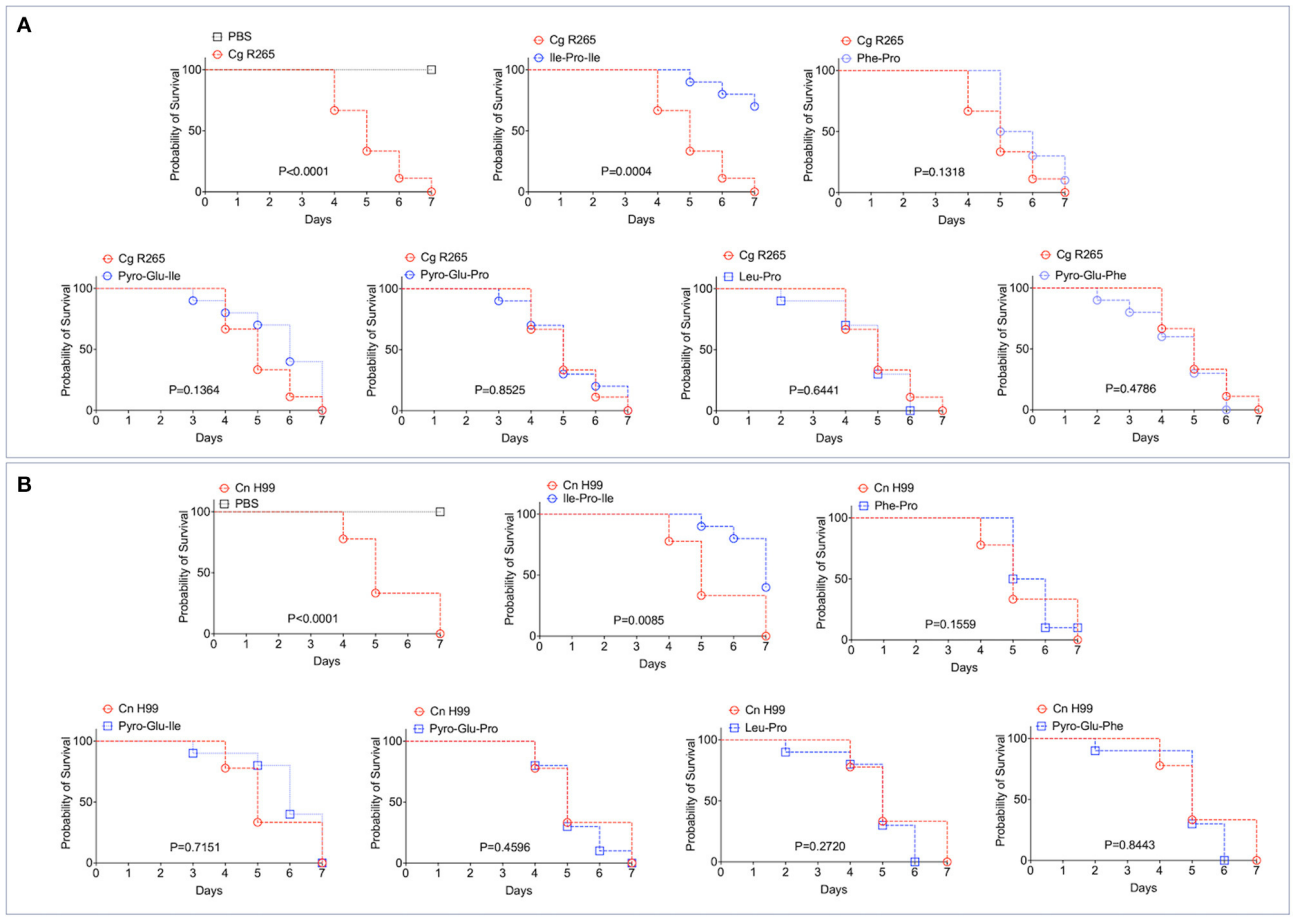
Effects of the EV peptides (1 μg/μl, equivalent to 10 μg per animal) on the survival of *G. mellonella* lethally infected with *C. gattii* R265 (Cg; **A**) or *C. neoformans* H99 (Cn; **B**). A. Ile-Pro-Ile was the only peptide prolonging the survival of *G. mellonella*. The other peptides did not interfere with the host's survival. The experiment illustrated in **(A)** was repeated using *C. neoformans* H99 instead of *C. gattii* R265, producing similar results. *P*-values resulting from the comparison of the survival curves were obtained using Log-rank Mantel-Cox test with the Graphpad Prism software, version 9.0. *P*-values lower than 0.05 represented significant statistical differences. Survival controls were obtained through injection of *G. mellonella* with PBS alone.

**Figure 5:**
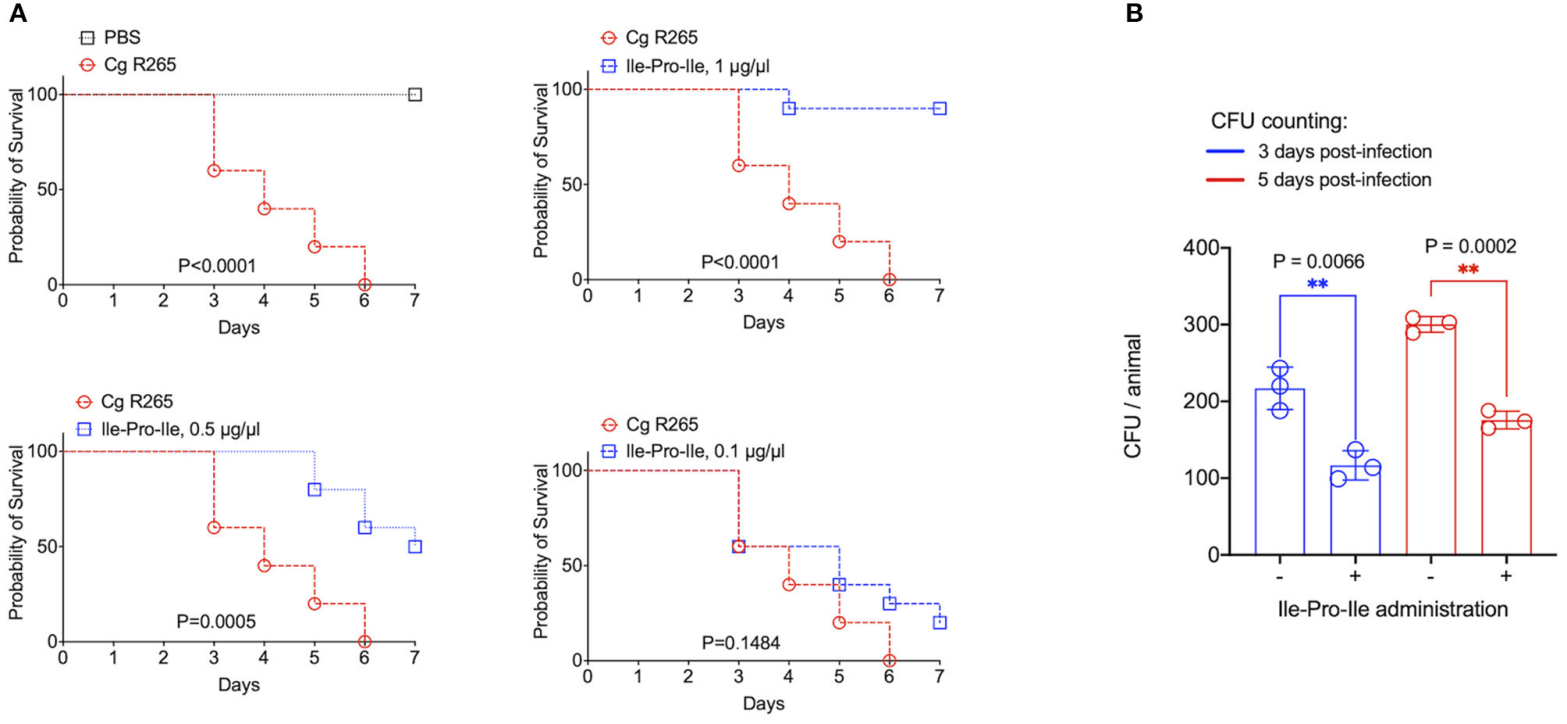
Protection of *G. mellonella* against *C. gattii* (Cg) induced by Ile-Pro-Ile. **(A)**. Survival of *G. mellonella* after injection with PBS alone (survival control) or with *C. gattii* yeast cells (left panel) is shown, in addition to the comparative survival curves of *G. mellonella* after injection with *C. gattii* alone (red curves) or with the fungus in the presence of variable concentrations of Ile-Pro-Ile. *P*-values resulting from the comparison of the survival curves were obtained using Log-rank Mantel-Cox test with the Graphpad Prism software, version 9.0. *P*-values lower than 0.05 represented significant statistical differences. **(B)**. Determination of fungal burden in infected larvae. Untreated and Ile-Pro-Ile-treated (1 μg/μl, equivalent to 10 μg per animal) infected larvae were macerated at days 3- and 5- post infection for CFU determination, which revealed a significantly decreased colonization of *G. mellonella* by *C. gattii* in peptide-treated systems (^**^). Paired statistical analysis after comparison between untreated and peptide-treated systems were performed using Student's *t*-test with the Graphpad Prism software, version 9.0.

It has been recently reported that fungal EVs, including cryptococcal vesicles, protect mice and the invertebrate host *Galleria mellonella* against lethal challenges with pathogenic fungi (12, 21–23). The vesicular molecules responsible for the protection remained unknown. We then asked whether the peptides listed in Table 2 could protect *G. mellonella* against a lethal challenge with *C. gattii*. We compared the mortality curves of *G. mellonella* infected with *C. gattii* alone with the mortality of the invertebrate host receiving *C. gattii* and each of the peptides at 1 μg/μl (equivalent to 10 μg per animal; Figure 4A). Phe-Pro, pyro-Glu-Ile, pyro-Glu-Pro, Leu-Pro, and pyro-Glu-Phe did not have any effect on the survival curves. In contrast, the tripeptide Ile-Pro-Ile significantly improved the survival of *G. mellonella*. We performed this experiment using *C. neoformans* instead of *C. gattii* and obtained similar results (Figure 4B). On the basis of these results, we selected Ile-Pro-Ile for tests at lower concentrations (1, 0.5, and 0.1 μg/μl, equivalent to 10, 5, and 1 μg per animal) in the *C. gattii* infection model. Once again, the peptide was highly efficient in prolonging the survival of lethally infected *G. mellonella* in a dose-dependent fashion (Figure 5A). The improved survival of lethally infected *G. mellonella* was accompanied by a significant reduction in the fungal burden, as concluded by counting colony forming units (CFU) in peptide-treated (10 μg per animal) and untreated larvae at 3- and 5-days post infection (Figure 5B).

## Discussion

The knowledge of the functions of fungal EVs has continuously increased in the recent years (7), but the biological roles of low mass structures exported in EVs are unknown. Small molecules secreted by *Cryptococcus* are immunologically active and affect IL-1β inflammasome-dependent secretion (24), but their association with EVs has not been established. In our study, we aimed at proving the concept that biologically active small molecules are exported in cryptococcal EVs. This idea culminated with the characterization for the first time of a fungal EV molecule inducing protection against pathogenic fungi.

Fungal EVs were demonstrated to mediate intercellular communication (4), prion transmission (25), biofilm formation associated with antifungal resistance (26), immunological responses *in vitro* (23, 27–30), and protection of different hosts against lethal challenges with fungal pathogens (12, 21–23). In any of these examples, these biological effects attributed to the EVs were correlated with the identification of the bioactive vesicular molecules. The only known exception was the protection of *G. mellonella* induced by cryptococcal EVs enriched with sterol glycosides and capsular polysaccharides (22). However, it is important to mention that the EVs in this study were produced by genetically engineered cells and, therefore, did not correspond to native vesicles. It remained also unknown if other molecules influenced the protective effects, since compositional studies have not been performed.

The identification of bioactive EV molecules is challenging in multiple aspects. The compositional analysis of fungal EVs in different models include a formidable variability in culture conditions, since each of the fungal pathogens tested so far manifest growth particularities. In this scenario, biomarkers of fungal EVs are still not known, although it has been suggested that mannoproteins and claudin-like Sur7 family proteins are important components of vesicles produced by *C. neoformans* and *C. albicans*, respectively (12, 31). The knowledge of small molecules mediating important biological activities in fungal EVs is even more limited. In *H. capsulatum*, carbohydrate metabolites were abundantly detected in EVs, in addition to L-ornithine and ethanolamine, among other small molecules (10). Noteworthy, in the *H. capsulatum* study, the conditions used for small molecule identification differed from those used in our study. Under similar conditions, we identified a comparable number of molecules in the EVs produced by the plant pathogen *P. digitatum* (11) and in *C. gattii* (this study). Specifically, small peptides were found in the EVs of these two distant species, reinforcing the notion that this molecular class is present in different fungal EVs. As for the possible detection of these molecules as artifacts in the *C. gattii* model, it is noteworthy that all identified peptides manifested high solubility in water, being susceptible to efficient removal by washing if they were not contained within the EVs. On the basis of their short sequences (2-4 amino-acids), the possibility that they will form insoluble, tertiary structures that will co-precipitate with EVs during ultracentrifugation is negligible.

Fungal toxins were also identified in *P. digitatum*, but this class of molecules is not normally produced by members the *Cryptococcus* genus. In *P. digitatum*, EVs were characterized as the carriers of tryptoquialanine A, a toxin that inhibited the germination of orange seeds (11). So far, tryptoquialanine A is the only low mass component of fungal EVs with a reported function. In this model, the mycotoxin fungisporin was also detected (11), but its function in fungal EVs remains to be determined. Together, these findings illustrate the need for an improved knowledge of the composition and functions of EV metabolites in fungi.

The isolation of cryptococcal EVs from solid medium is much more efficient than the similar protocols using liquid cultures (5). RNA and proteins in cryptococcal EVs obtained in liquid cultures were characterized in early studies (8, 32), but their distribution in EVs obtained from solid medium was only recently described in *C. neoformans* (12). Other molecules remained unknown, and the metabolite composition of cryptococcal EVs has not been investigated so far. In our study, we initially aimed at understanding what are the low molecular weight components exported by *C. gattii* in solid medium. We identified small molecules of different chemical natures as putative components of cryptococcal EVs, but their functions remain widely unknown. However, our chemical and biological methods for structural validation revealed that one tripeptide was capable to protect *G. mellonella* against lethal challenges with *C. gattii* or *C. neoformans*. The mechanisms by which the peptides induced protection against cryptococcal infection remain unknown, but the immune response of *G. mellonella* is innate and relies on the activity of hemocytes in combination with antimicrobial peptides and lytic enzymes, among others (33). Accordingly, immunity to *Cryptococcus* relies on innate immune cells coordinating adaptive responses to stimulate fungal killing (34). Therefore, we hypothesize that the tripeptide identified in our study is an inducer of innate responses, which have a key general role in the control of fungal infections (35). Other possibilities, however, cannot be ruled out, as follows below.

The peptide inducing protection against *Cryptococcus* in *G. mellonella* was demonstrated to have important biological activities in other models. Ile-Pro-Ile, also known as diprotin A, is an inhibitor of dipeptidyl peptidase 4, an enzyme participating in insulin metabolism (36) and chemotaxis of murine embryonic stem cells toward stromal cell-derived factor-1 (37). Its role in fungal physiology and/or pathogenesis was also suggested. In *Aspergillus fumigatus*, a dipeptidyl peptidase 4 was purified from fungal cultures and a role in binding to collagen and activation of CD4+ T cells was speculated (38). It was also reported that *Blastomyces dermatitidis* produces dipeptidyl peptidase 4. In this model, the enzyme was responsible for disabling innate immunity mechanisms and promoting pathogenicity (39). If a similar mechanism is functional in the *Cryptococcus* model, free Ile-Pro-Ile administered in the *G. mellonella* infection model could inhibit the fungal dipeptidyl peptidase 4 with a consequently decreased pathogenicity. Noteworthy, our study did not elucidate any physiological or pathogenic functions. Instead, we present a proof of concept that fungal EVs are the vehicles for exporting biologically active molecules of low molecular mass that may be involved in immunological and/or pathogenic mechanisms. Since fungal EVs have been consistently proposed as vaccine candidates in different models, the potential of these findings can be substantial.

## Methods

### Preparation of EVs

The EV-producing isolate used in this study was the standard strain R265 of *C. gattii*. Of note, the R265 strain has been recently reclassified as *C. deuterogattii* (40). In this study, we kept its classification as *C. gattii*, as largely employed in the *Cryptococcus* literature. EV isolation was based on the protocol that we have recently established for *C. gattii* and other fungal species (5). Briefly, One colony of *C. gattii* R265 cultivated in solid Sabouraud's medium was inoculated into yeast extract-peptone-dextrose (YPD) medium (5 ml) and cultivated for 1 day at 30°C with shaking. The cell density was adjusted to of 3.5 × 10^7^cells/ml in YPD. From this suspension, aliquots of 300 μl were taken for inoculation in YPD agar plates, which were cultivated for 1 day at 30°C. The cells were recovered from the plates with an inoculation loop and transferred to a single centrifuge tube containing 30 ml of PBS filtered through 0.22-μm-pore membranes. The cells were then removed by centrifugation (5,000 × g for 15 min at 4°C), and the supernatants were centrifuged again (15,000 × g for 15 min at 4°C) to remove debris. The resulting supernatants were filtered through 0.45-μm-pore syringe filters and again centrifuged (100,000 × g, 1 h at 4°C). Supernatants were discarded and pellets suspended in 300 μl of sterile PBS. To avoid the characterization of medium components as EV molecules, mock (control) samples were similarly prepared using sterile plates containing YPD. Four petri dishes were used for each EV isolation, and EV isolation was performed independently three times. In all samples, the properties of EVs and their concentration were monitored by nanoparticle tracking analysis (NTA) and transmission electron microscopy as described by our group (5). The samples prepared for mass spectrometry analyses had the typical properties of *C. gattii* EVs, and were in the range of 4–6 × 10^10^ EVs within the triplicate set.

### Mass Spectrometry Analyses

*C. gattii* EVs were vacuum dried and extracted with 1 ml of methanol during 1 h in an ultrasonic bath. The extracts were filtered (0.22 μm), dried under a gentle N_2_ flux and stored at −20°C. EV extracts were resuspended in 200 μl of MeOH and transferred into glass vials. Ultra high-performance liquid chromatography-mass spectrometry (UHPLC-MS) analyses were performed using a Thermo Scientific QExactive® hybrid Quadrupole-Orbitrap mass spectrometer with the following parameters: electrospray ionization in positive mode, capillary voltage at 3.5 kV; capillary temperature at 300°C; S-lens of 50 V and *m/z* range of 100.00–1500.00. Tandem Mass spectrometry (MS/MS) was performed using normalized collision energy (NCE) of 20, 30, and 40 eV; maximum 5 precursors per cycle were selected. Stationary phase was a Waters ACQUITY UPLC® BEH C18 1.7 μm (2.1 × 50 mm) column. Mobile phases were 0.1% (v/v) formic acid in water (A) and acetonitrile (B). Eluent profile (A:B) 0–10 min, gradient from 95:5 up to 2:98; held for 5 min; 15–16.2 min gradient up to 95:5; held for 3.8 min. Flow rate was 0.2 mL min^−1^. Injection volume was 3 μL. UHPLC-MS operation and spectra analyses were performed using Xcalibur software (version 3.0.63).

### Molecular Network

A molecular network was created using the online workflow (https://ccms-ucsd.github.io/GNPSDocumentation/) on the GNPS website (http://gnps.ucsd.edu). The data was filtered by removing all MS/MS fragment ions within ±17 Da of the precursor m/z. MS/MS spectra were window filtered by choosing only the top 6 fragment ions in the ±50 Da window throughout the spectrum. The precursor ion mass tolerance was set to 0.02 Da and a MS/MS fragment ion tolerance of 0.02 Da. A network was then created where edges were filtered to have a cosine score above 0.5 and more than 5 matched peaks. Further, edges between two nodes were kept in the network if and only if each of the nodes appeared in each other's respective top 10 most similar nodes. Finally, the maximum size of a molecular family was set to 100, and the lowest scoring edges were removed from molecular families until the molecular family size was below this threshold. The spectra in the network were then searched against GNPS' spectral libraries. The library spectra were filtered in the same manner as the input data. All matches kept between network spectra and library spectra were required to have a score above 0.5 and at least 5 matched peaks (14).

### Peptides

The peptides selected for biological tests were synthesized by GenOne Biotechnologies (https://www.genone.com.br, Rio de Janeiro, Brazil). Purity and structural properties of each peptide were confirmed by high-performance liquid chromatography coupled to mass spectrometry. All peptides were water-soluble and had their purity at the 95% range.

### *Galleria mellonella* Infection Model

Groups of 10 larvae (250–350 mg) were used for injection into the last left proleg using a Hamilton micro-syringe. The injection systems (10 μl) consisted of sterile PBS alone, sterile PBS containing 10^6^ cells of *C. gattii* or *C. neoformans*, or sterile PBS containing *C. gattii* or *C. neoformans* and Ile-Pro-Ile, Phe-Pro, pyro-Glu-Ile, pyro-Glu-Pro, Leu-Pro, and pyro-Glu-Phe at 1 μg/μl (equivalent to 10 μg per animal). Due to its promising effects, Ile-Pro-Ile was also tested at 0.5, and 0.1 μg/μl (equivalent to 5 μg and 1 per animal) in a *C. gattii* model of infection. Injected larvae were placed in sterile Petri dishes and incubated at 37°C. The survival was monitored daily in a period of 7 days. Larvae were considered dead if they did not respond to physical stimulus. Statistical analysis in the survival curves was performed using the Log-rank Mantel-Cox test with the Graphpad Prism software, version 9.0. Infected larvae were also used for determination of fungal burden. Additional experimental sets were prepared as described for survival curves, but the experiments were interrupted at days 3- and 5- post infection. Ile-Pro-Ile concentration in these assays corresponded to 1 μg/μl. Surviving larvae (*n* = 5 at day 3-post infection; *n* = 4 at day 5-post infection) were macerated in 3 ml PBS and 100 μl were plated onto Sabouraud agar plates supplemented with 1% penicillin and streptomycin. The plates were incubated at 30°C for 48 h for further CFU counting. Paired statistical analysis after comparison between untreated and peptide-treated systems were performed using Student's *t*-test with the Graphpad Prism software, version 9.0.

## Data Availability Statement

The raw data supporting the conclusions of this article will be made available by the authors, without undue reservation.

## Author Contributions

FR, JC, and LH performed the experiments. LN, TF, and MR raised funds, interpreted the data, and wrote the manuscript. All authors contributed to the article and approved the submitted version.

## Conflict of Interest

The authors declare that the research was conducted in the absence of any commercial or financial relationships that could be construed as a potential conflict of interest.
