# Supplementary material for: Small Molecule Analysis of Extracellular Vesicles Produced by Cryptococcus gattii: Identification of a Tripeptide Controlling Cryptococcal Infection in an Invertebrate Host Model
Source: Front Immunol. 2021 Mar 16;12:654574. doi: 10.3389/fimmu.2021.654574 (PMC8008140; doi:10.3389/fimmu.2021.654574)
Supplement: Supplementary file 1 [file Data_Sheet_1.pdf]

Supplemental material

Supplemental Figures 1-13. Metabolite identification through comparison between the MS data obtained in our study with hits in the GNPS database. Mass spectrometry (MS) profiles are shown for Ile-Pro-Ile (Figure S1), Phe-Pro (Figure S2), pyro-Glu-Ile (Figure S3), pyro-Glu-Pro (Figure S4), Leu-Pro (Figure S5), pyro-Glu-Phe (Figure S6), Val-Leu-Pro-Val-Pro (Figure S7), cyclo(Trp-Pro) (Figure S8), cyclo(Tyr-Pro) (Figure S9), tryptophan (Figure S10), asperphenamate (Figure S11), riboflavin (Figure S12), and pantothenic acid (Figure S13).

Supplemental Figure 14. Structural aspects of the main mass fragments obtained for the amino acids leucine, proline and phenylalanine.

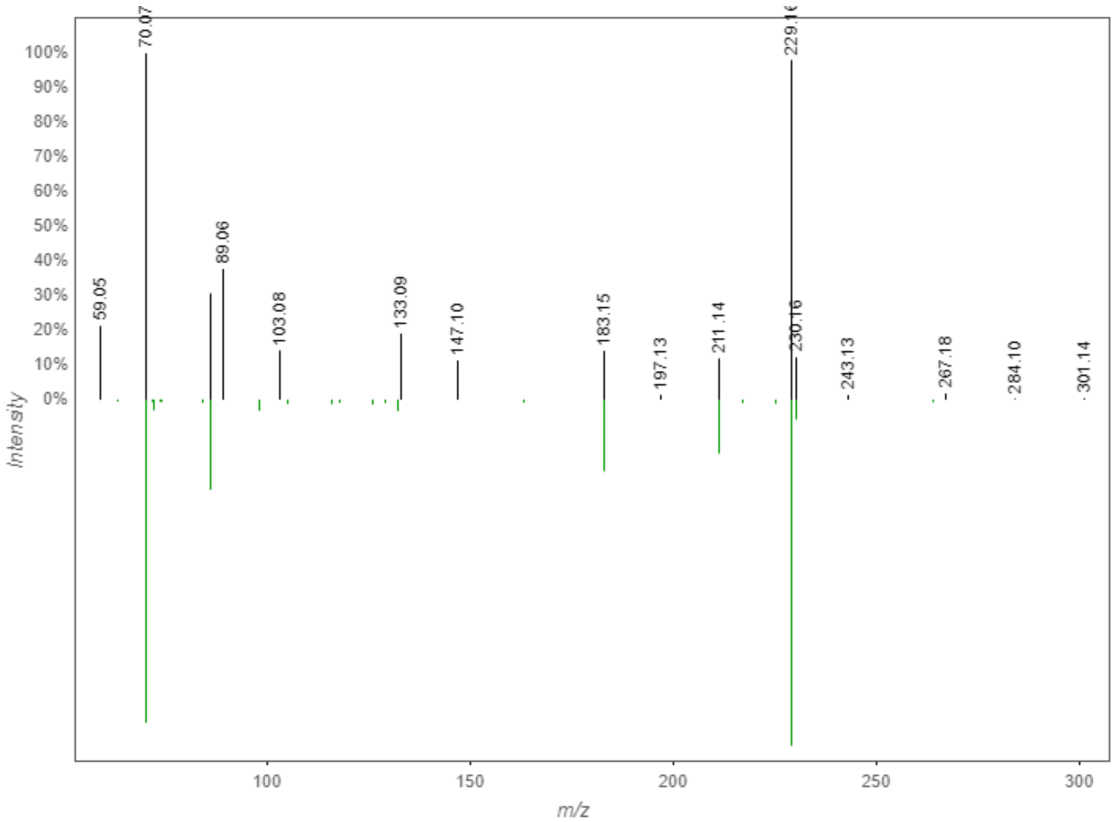

Figure S1. GNPS hit/MS data for Ile-Pro-Ile.

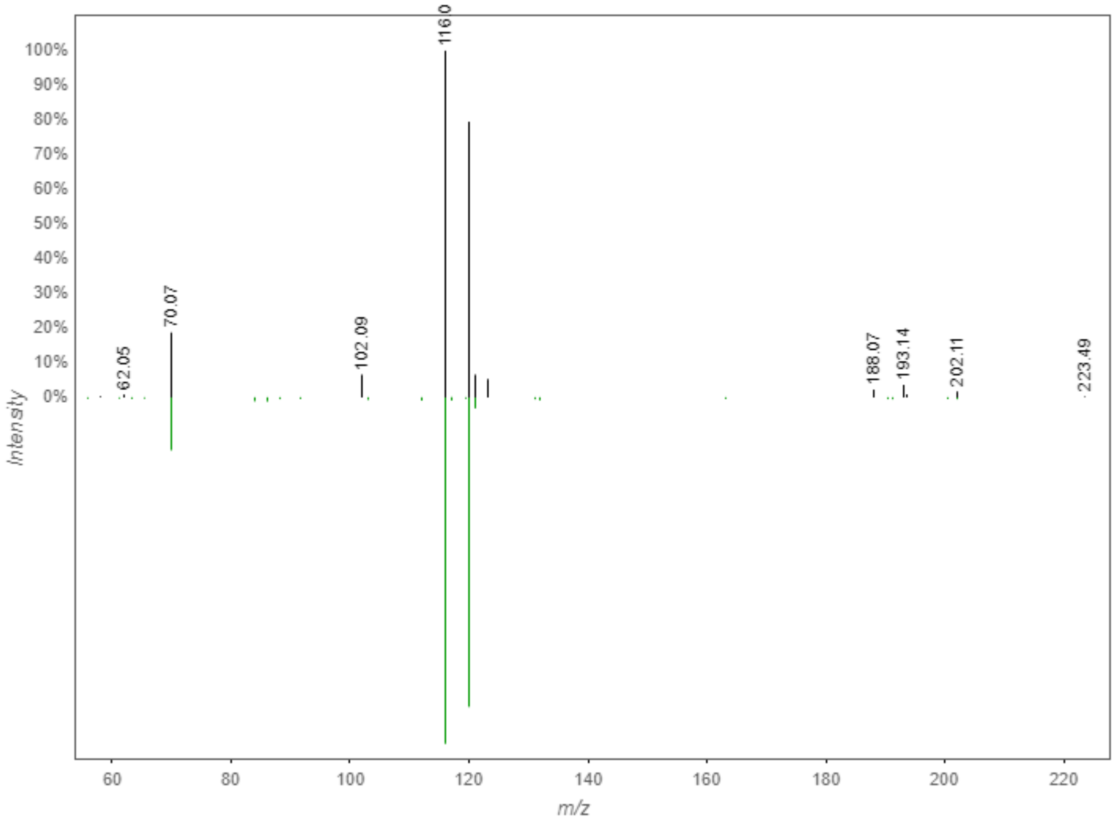

Figure S2. GNPS hit/MS data for Phe-Pro.

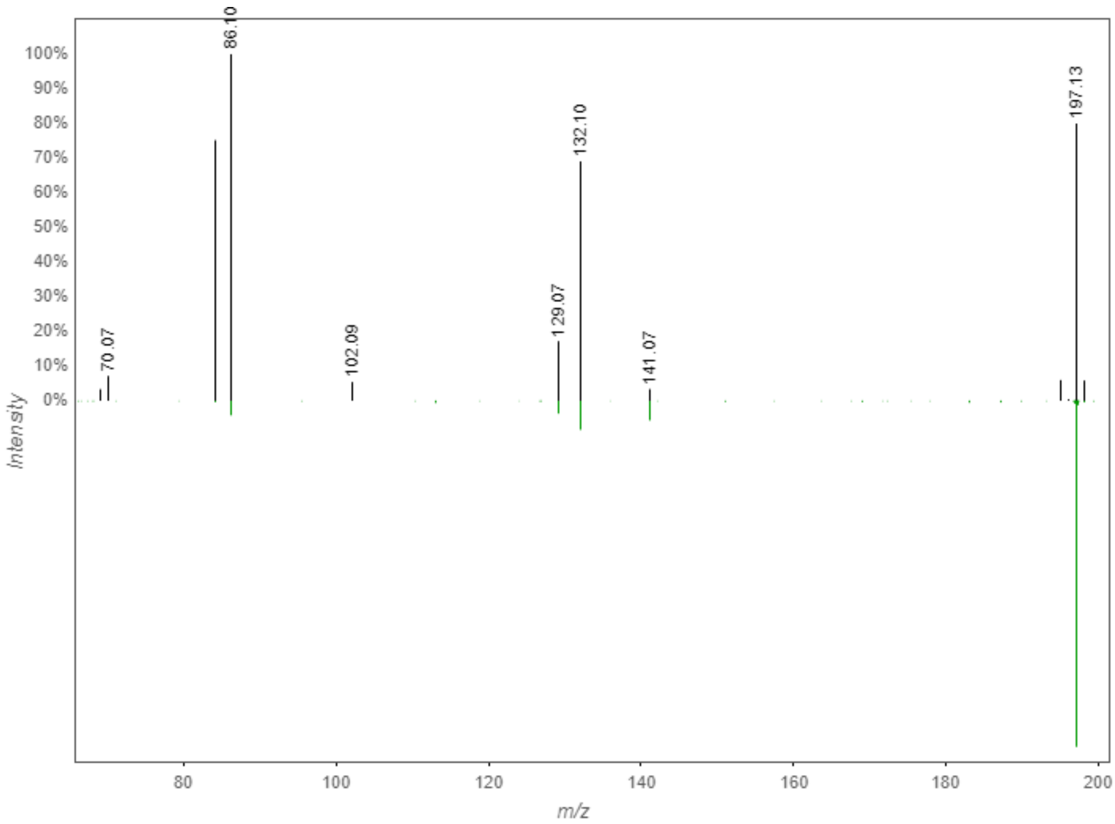

Figure S3. GNPS hit/MS data for pyro-Glu-Ile.

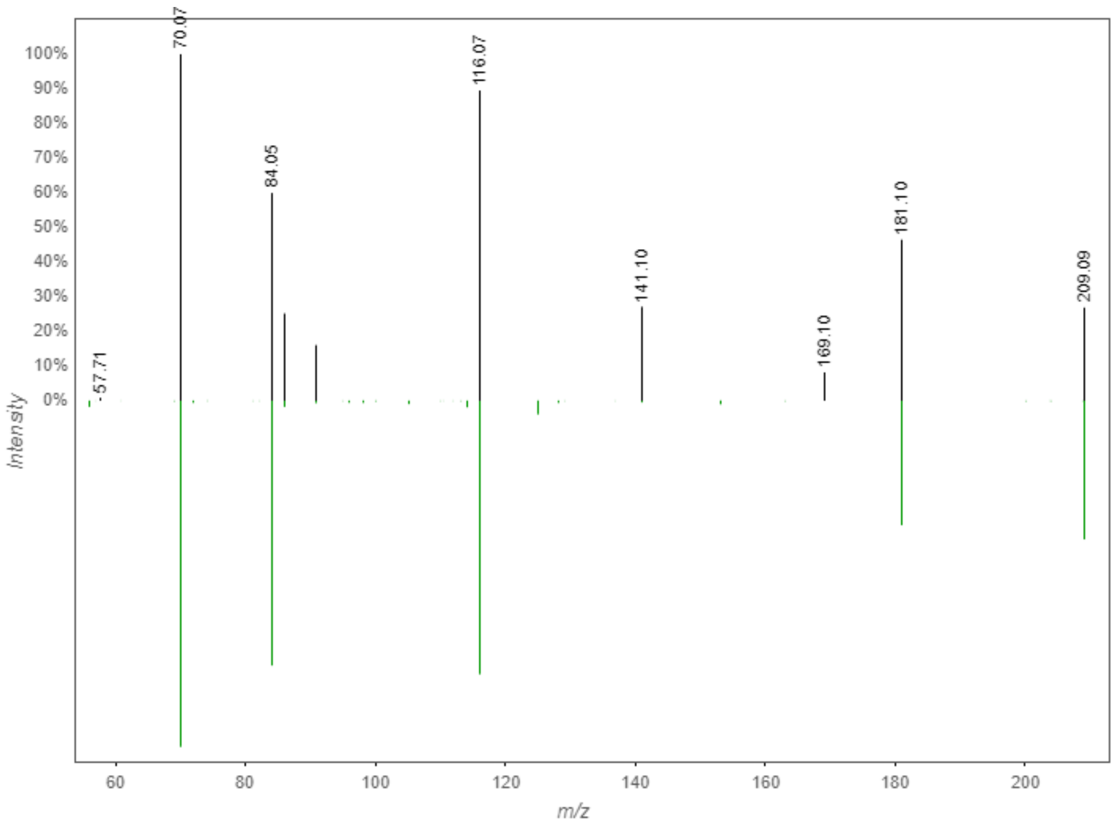

Figure S4. GNPS hit/MS data for pyro-Glu-Pro.

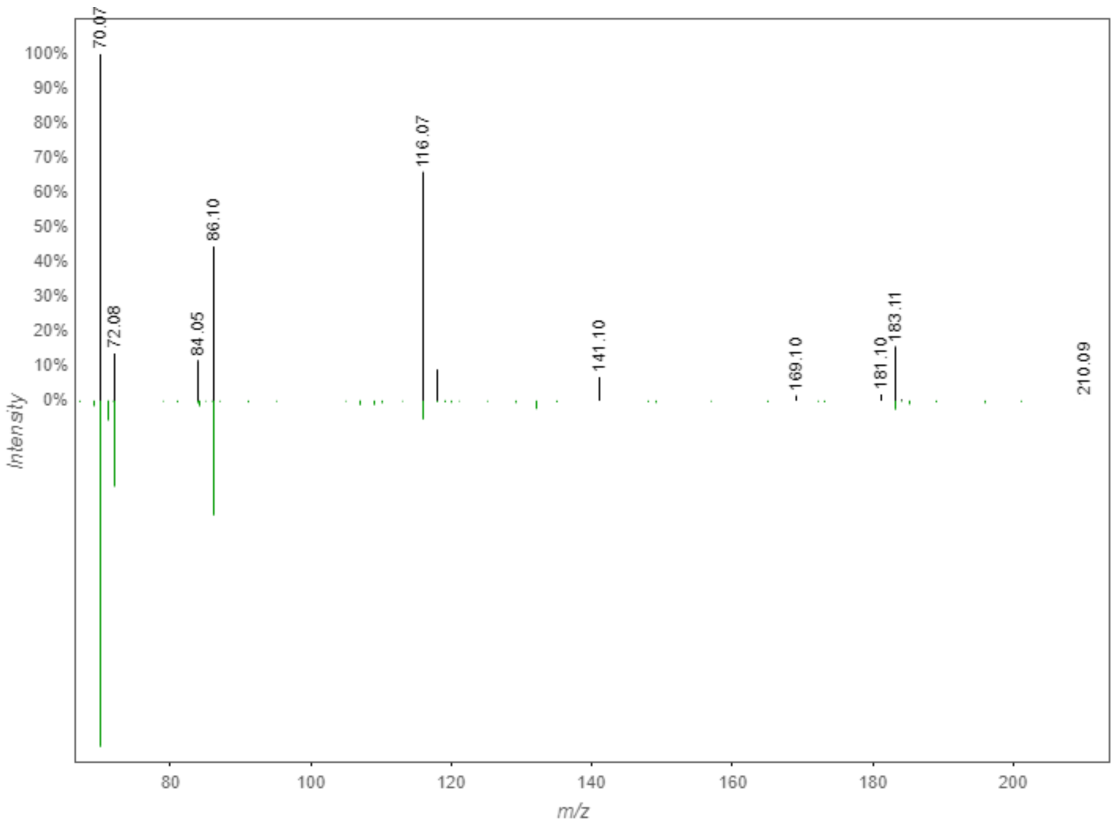

Figure S5. GNPS hit/MS data for Leu-Pro.

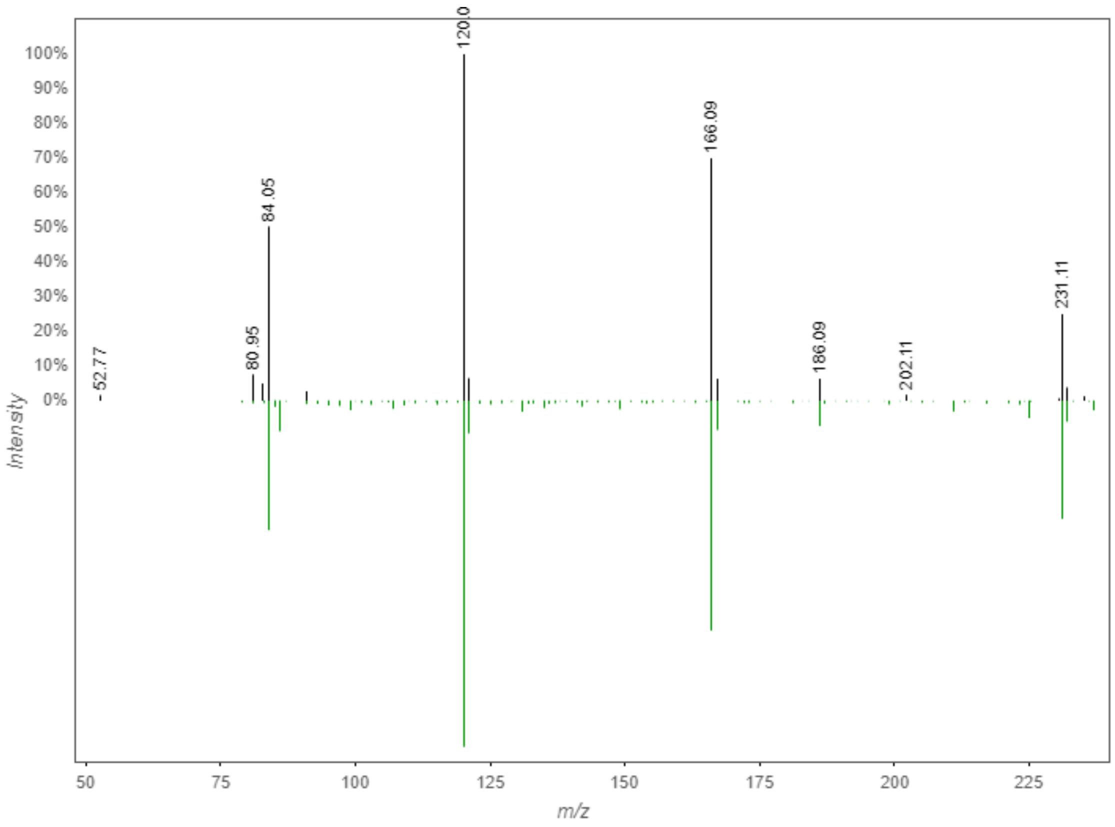

Figure S6. GNPS hit/MS data for pyro-Glu-Phe.

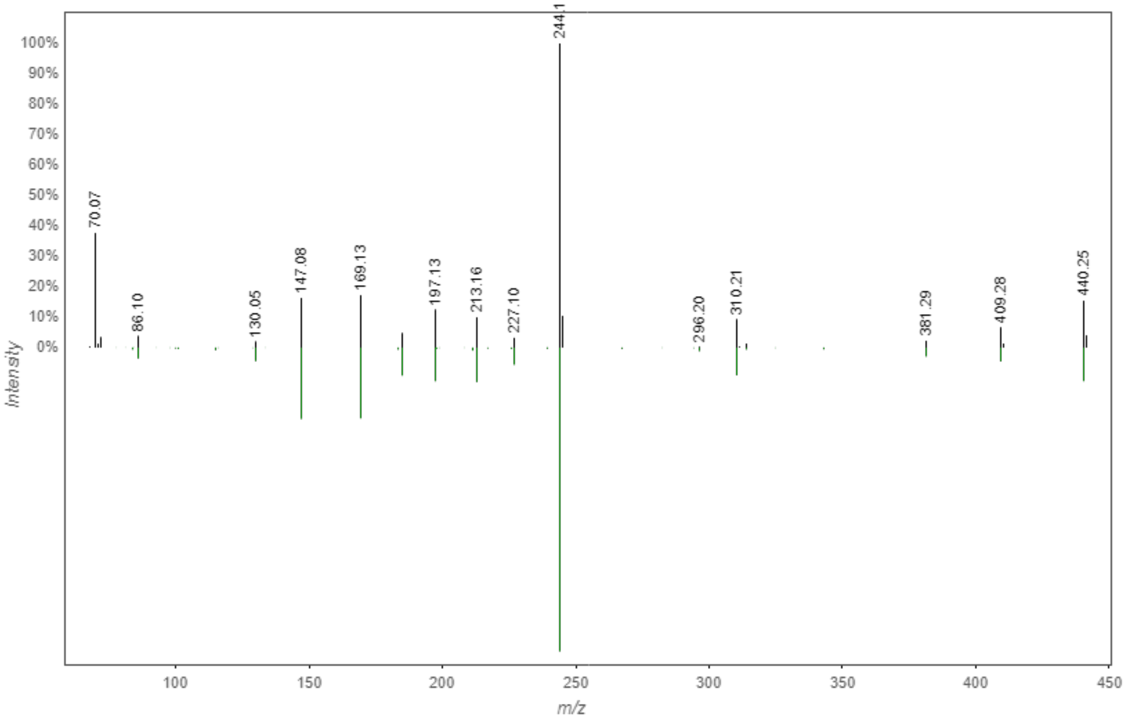

Figure S7. GNPS hit/MS data for Val-Leu-Pro-Val-Pro.

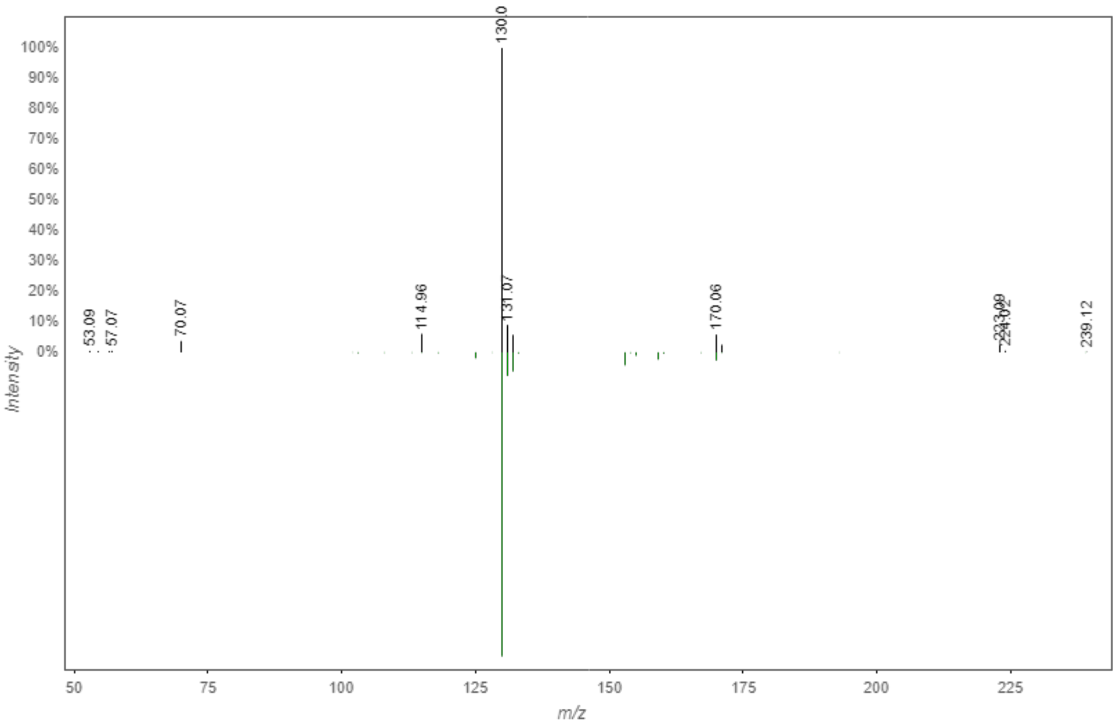

Figure S8. GNPS hit/MS data for cyclo(Trp-Pro).

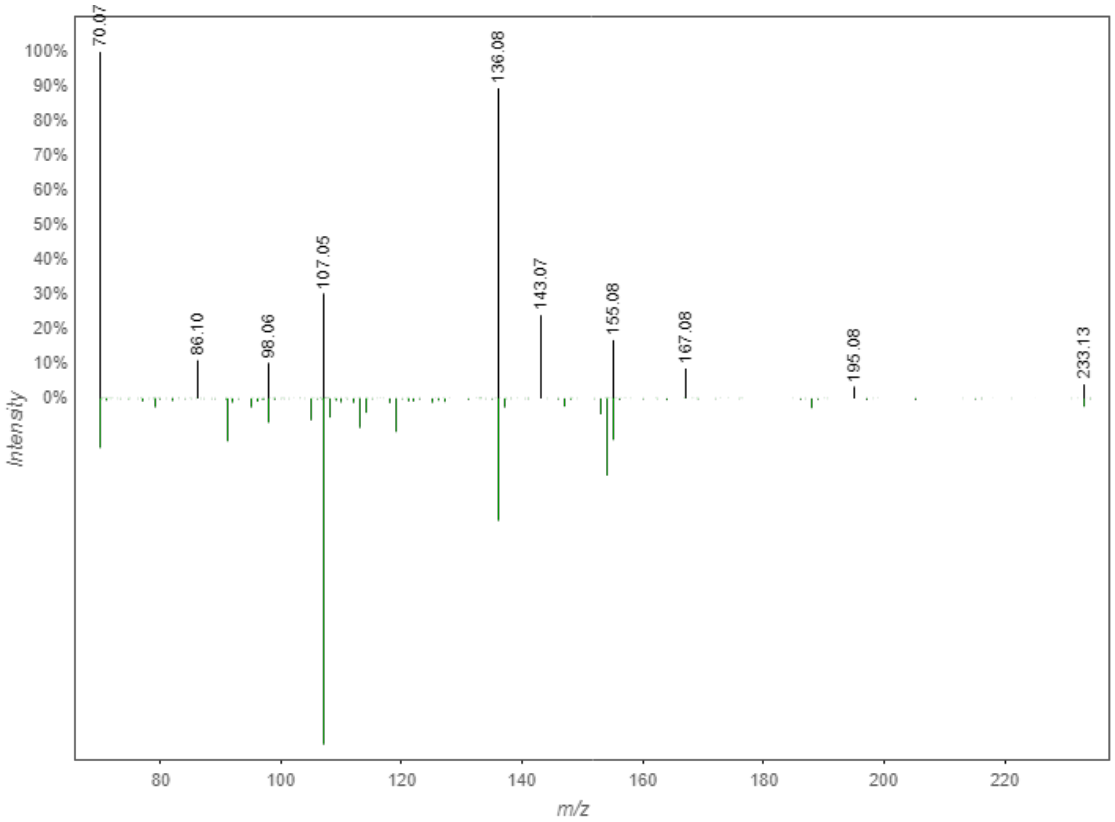

Figure S9. GNPS hit/MS data for cyclo(Tyr-Pro).

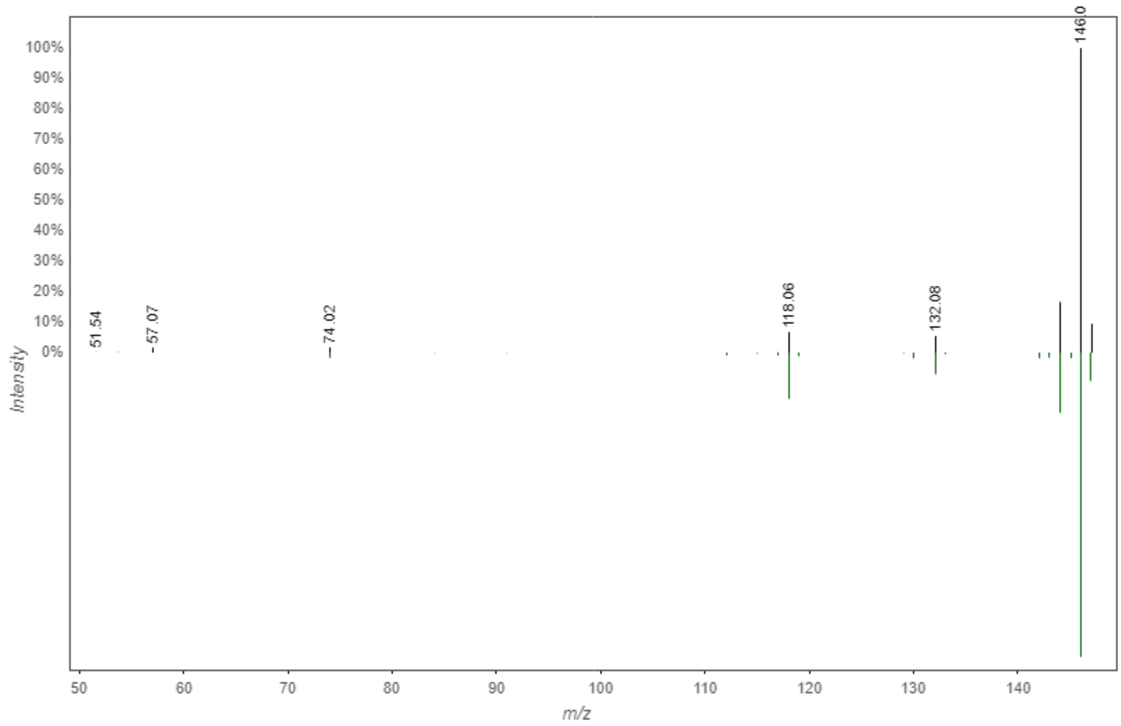

Figure S10. GNPS hit/MS data for tryptophan.

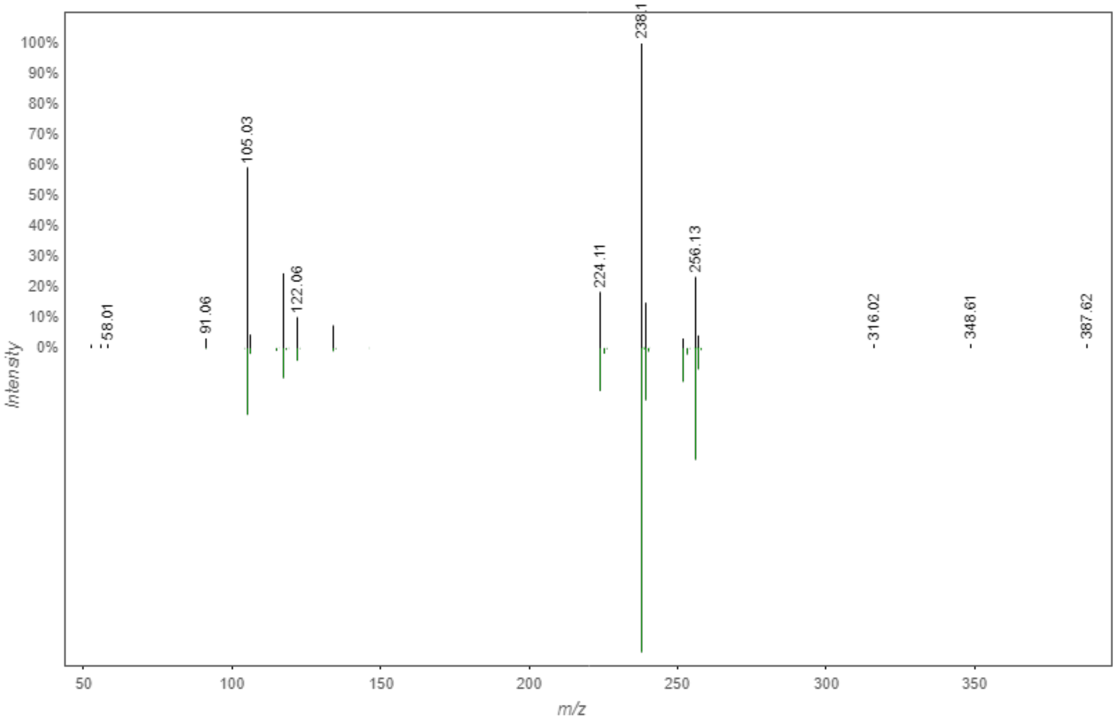

Figure S11. GNPS hit/MS data for asperphenamate.

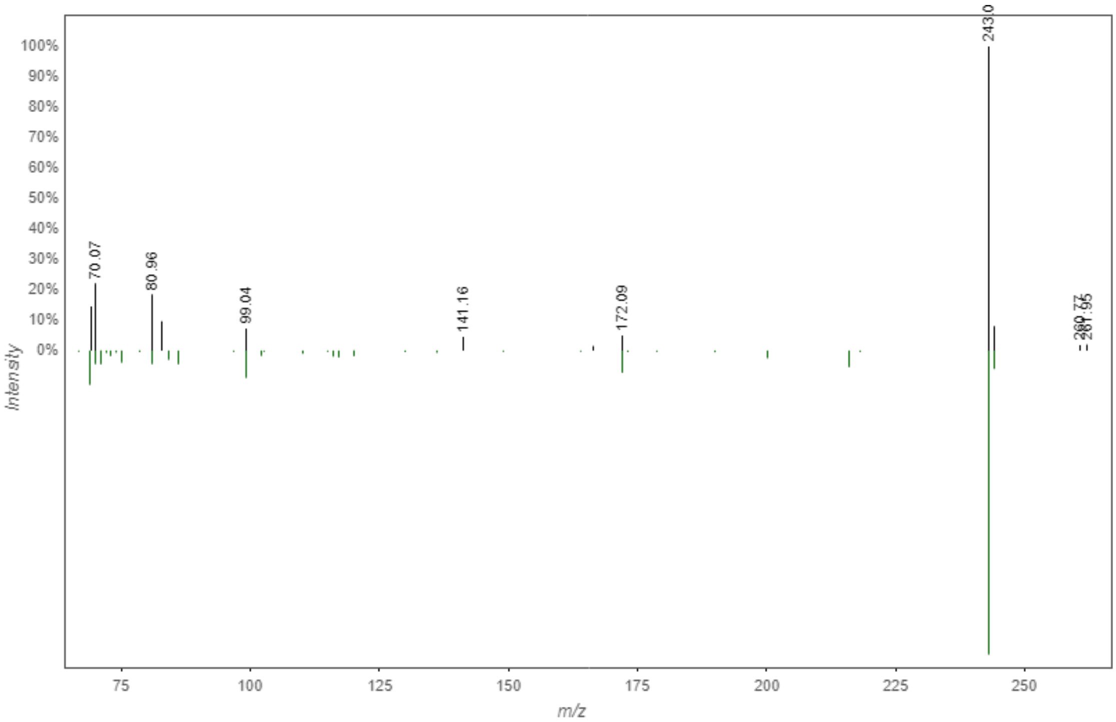

Figure S12. GNPS hit/MS data for riboflavin.

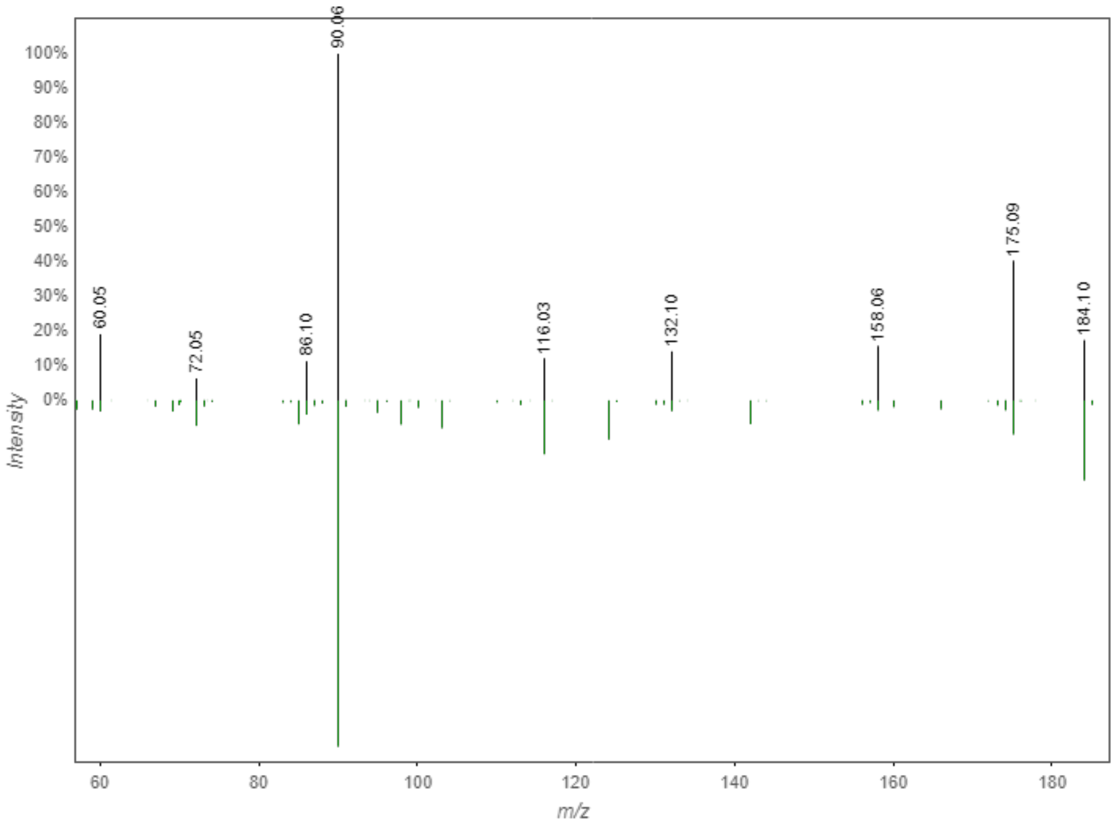

Figure S13. GNPS hit/MS data for pantothenic acid.

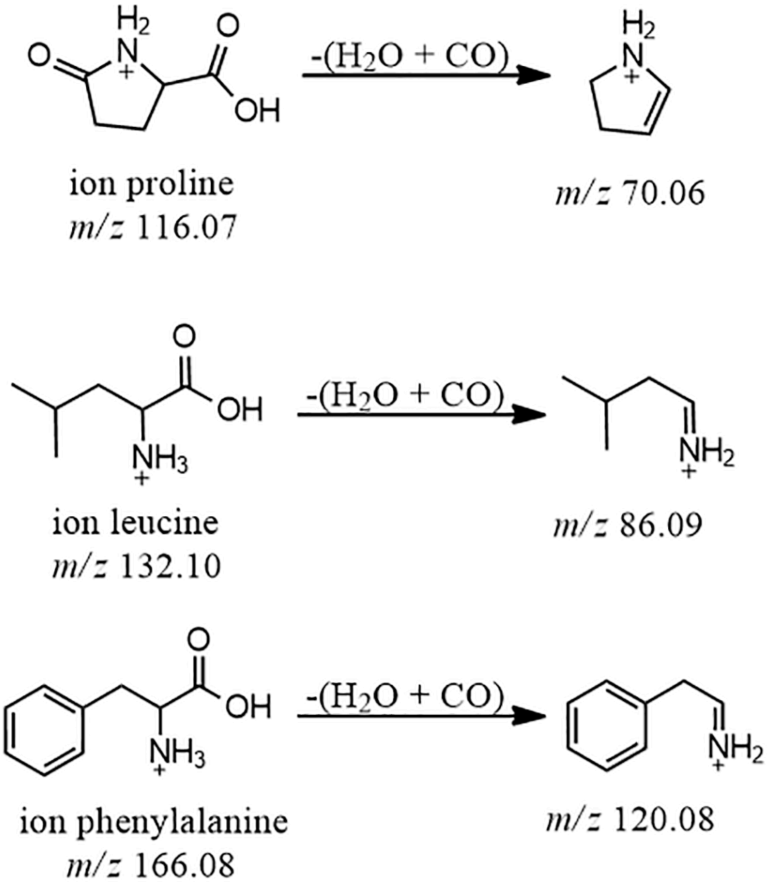

Figure S14. Main mass fragments obtained for amino acids leucine, proline and phenylalanine.
